# BIM mediates oncogene inactivation-induced apoptosis in multiple transgenic mouse models of acute lymphoblastic leukemia

**DOI:** 10.18632/oncotarget.8731

**Published:** 2016-04-14

**Authors:** Yulin Li, Anja Deutzmann, Peter S. Choi, Alice C. Fan, Dean W. Felsher

**Affiliations:** ^1^ Division of Oncology, Department of Medicine and Pathology, Stanford University, Stanford, CA, United States of America

**Keywords:** apoptosis, BIM, targeted therapies, oncogene inactivation

## Abstract

Oncogene inactivation in both clinical targeted therapies and conditional transgenic mouse cancer models can induce significant tumor regression associated with the robust induction of apoptosis. Here we report that in MYC-, RAS-, and BCR-ABL-induced acute lymphoblastic leukemia (ALL), apoptosis upon oncogene inactivation is mediated by the same pro-apoptotic protein, BIM. The induction of BIMin the MYC- and RAS-driven leukemia is mediated by the downregulation of *miR-17-92*. Overexpression of *miR-17-92* blocked the induction of apoptosis upon oncogene inactivation in the MYC and RAS-driven but not in the BCR-ABL-driven ALL leukemia. Hence, our results provide novel insight into the mechanism of apoptosis upon oncogene inactivation and suggest that induction of BIM-mediated apoptosis may be an important therapeutic approach for ALL.

## INTRODUCTION

Although cancers evolve through a multistage process with accumulation of both genetic and epigenetic changes, many cancers are dependent on specific driver oncogenes for maintenance of the malignancy [1]. This phenomenon of oncogene dependence, termed “oncogene addiction”, provides the rationale for targeted therapy of human cancers.

The inactivation of driver oncogenes in mouse cancer models and human targeted therapy often leads to tumor regression associated with the induction of apoptosis [2–5]. The mechanism by which oncogene inactivation induces apoptosis in cancer has not been defined. Apoptosis occurs through two pathways: the extrinsic and intrinsic pathways. The activation of the intrinsic apoptosis pathway is determined by the balance between the pro-apoptotic BCL-2 family proteins BIM, BAD, BAX, and PUMA, and the anti-apoptotic proteins BCL-2, BCL-xL, and MCL1 [6–8]. BIM-mediated apoptosis is critical for the development and homeostasis of immune cells [9]. *Bim* deficient mice often develop autoimmune diseases, and lymphocytes from these mice are refractory to apoptotic stimuli. More recently, BIM, together with other BCL-2 family proteins, have been implicated in the mechanism of apoptosis and therapeutic sensitivity of BCR-ABL positive cells treated with imatinib, lung adenocarcinoma cells treated with EGFR inhibitors, and breast cancer cells treated with HER2 inhibitors [10–18].

**Figure 1:**
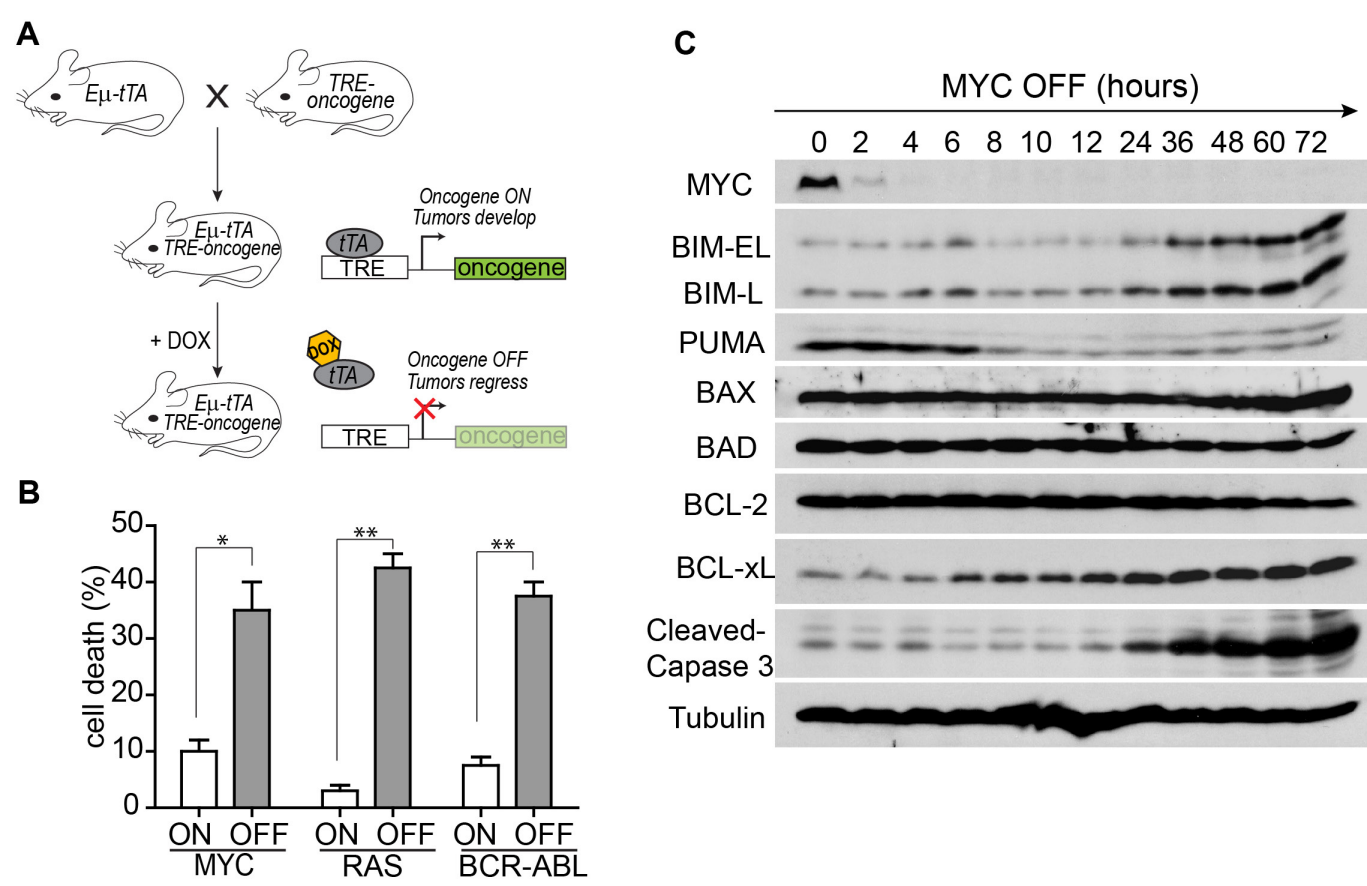
Apoptosis induction upon oncogene inactivation in Tet-regulated mouse ALL models **A.** The ALL mouse models controlled by the Tet-off system. In the absence of doxycycline (DOX), tTA binds to TRE to drive the overexpression of the oncogenes. In the presence of DOX, tTA cannot bind and oncogene expression is off. **B.** Inactivation of the oncogenes induced massive apoptosis of tumor cells according to 7-AAD staining. Oncogene expression was shutoff for three days in the MYC and RAS models and for one day in the BCR-ABL model. Each time point includes three replicates. Data are presented as mean +/− SEM. Student's *t* test. *p < 0.01, **p < 0.001. **C.** Expression of BCL-2 family proteins and caspase 3 cleavage upon the inactivation of MYC. Protein samples were collected every two hours for the first 12 hours and then every 12 hours.

Oncogenes, such as *MYC, RAS*, and *BCR-ABL*, are frequently involved in the pathogenesis of human acute lymphoblastic leukemia (ALL) [19]. We and others have developed multiple conditional transgenic mouse models of ALL using the Tet-off system [3, 4]. The immunoglobulin heavy chain enhancer (*Eμ*) is used to regulate the expression of Tet transactivator (tTA) in lymphocytes. By crossing of *Eμ*-tTA mice with mice carrying different oncogenes controlled by a tetracycline-responsive element (*TRE*), we are able to regulate oncogene expression in lymphocytes (Figure 1A). *Eμ-tTA/TRE-MYC* and *Eμ-tTA/TRE-RAS* mice develop T-cell ALL, while *Eμ-tTA/TRE-BCR-ABL* mice develop B-cell ALL [3, 4, 20]. Upon oncogene inactivation by administering doxycycline, the leukemia undergoes dramatic regression associated with proliferative arrest, senescence, and apoptosis [3, 4]. Here we have used these transgenic mouse models driven by different oncogenes to investigate the mechanism by which oncogene inactivation induces apoptosis.

## RESULTS

### BIM expression is induced by oncogene inactivation in MYC-, RAS-, and BCR-ABL-driven ALL leukemia models

**Figure 2:**
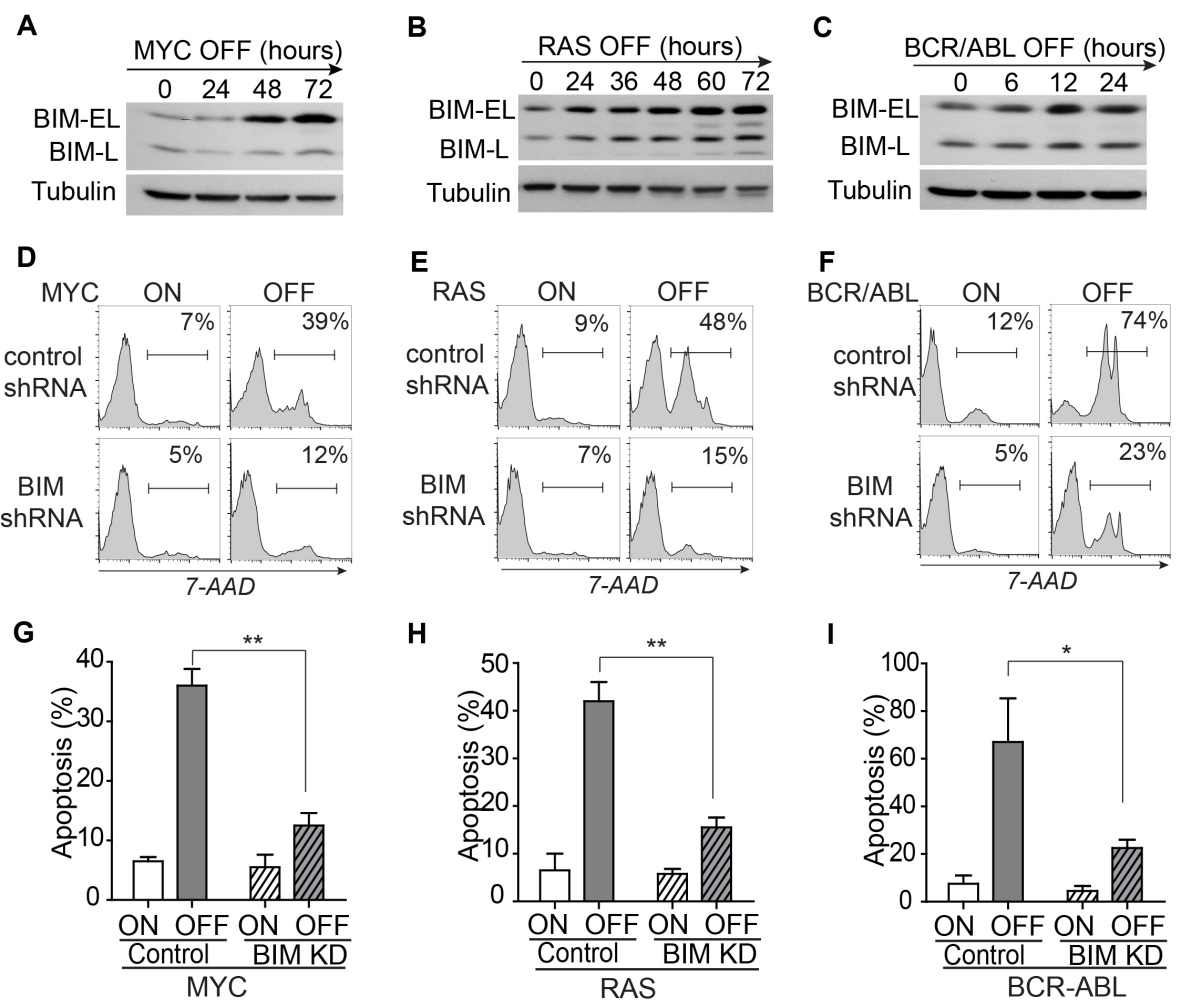
BIM mediates apoptosis induced by inactivation of the driver oncogene **A.**-**C.** The gradual induction of *Bim* protein expression upon the inactivation of the MYC, RAS, and BCR-ABL. Both BIM-EL and BIM-L isoforms are shown. The level of tubulin served as loading control. **D.**-**F.** Knockdown of BIM expression blocks the induction of apoptosis by oncogene inactivation. Oncogene expression was shutoff for three days in the MYC and RAS models and for two days in the BCR-ABL model. The gates for 7-AAD positive populations are set as indicated. Apoptosis rates are labeled in the top right corners of the plots. For each cell line, only one representative plot is shown. Data for the replicates was further quantified and presented in figure G-I. **G.**-**I.** Quantification of the apoptosis rates in control *versus* tumor cells with BIM knockdown. Each time point includes three replicates. Data are presented as mean +/− SEM. Student's *t* test. Data are presented as mean +/− SEM. Student's *t* test. *p < 0.05, **p < 0.001.

Tumor derived cell lines were generated from the MYC-, RAS-, and BCR-ABL-induced ALLs. Upon oncogene inactivation with doxycycline, the *E*μ*-tTA/TRE-MYC* leukemia cells underwent significant cell death as shown by 7-AAD staining (Figure 1B). To determine the mechanism of apoptosis, we measured the level of many BCL-2 family proteins by Western blot analysis. The level of BAD, BAX, and BCL-2 did not change, whereas PUMA decreased upon MYC inactivation (Figure 1C). BIM was the only pro-apoptotic protein induced and temporally associated with activation of Caspase 3. The level of BIM gradually increased starting at 24 hours of MYC inactivation and was about ten-fold higher at 72 hours (Figure 2A). Similarly, BIM protein increased eight-fold upon RAS inactivation and three-fold upon BCR-ABL inactivation in the *E*μ *-tTA/TRE-RAS* and *E*μ*-tTA/TRE-BCR-ABL* leukemia, respectively (Figure 2B–2C). Interestingly, the timing of BIM induction corresponded to the timing of apoptosis induction in all three tumor types. These data suggest that BIM may be a common mediator of apoptosis induction upon oncogene inactivation.

### Suppression of BIM blocks the induction of apoptosis and impedes tumor regression

Next, we examined whether the shRNA-mediated knockdown of BIM expression influenced the induction of apoptosis. A *miR-30*-based shRNA was used to knockdown BIM expression by 80% as measured by Western blot analysis [21] (Supplementary Figure 1). BIM** knockdown blocked apoptosis upon inactivation of the driver oncogenes (MYC 39% *versus* 12%; RAS 48% *versus* 15%; BCR-ABL 74% *versus* 23%; as shown in Figure 2D–2F and further quantified in Figure 2G–2I). Thus, suppression of BIM expression blocks the induction of apoptosis upon oncogene inactivation.

**Figure 3:**
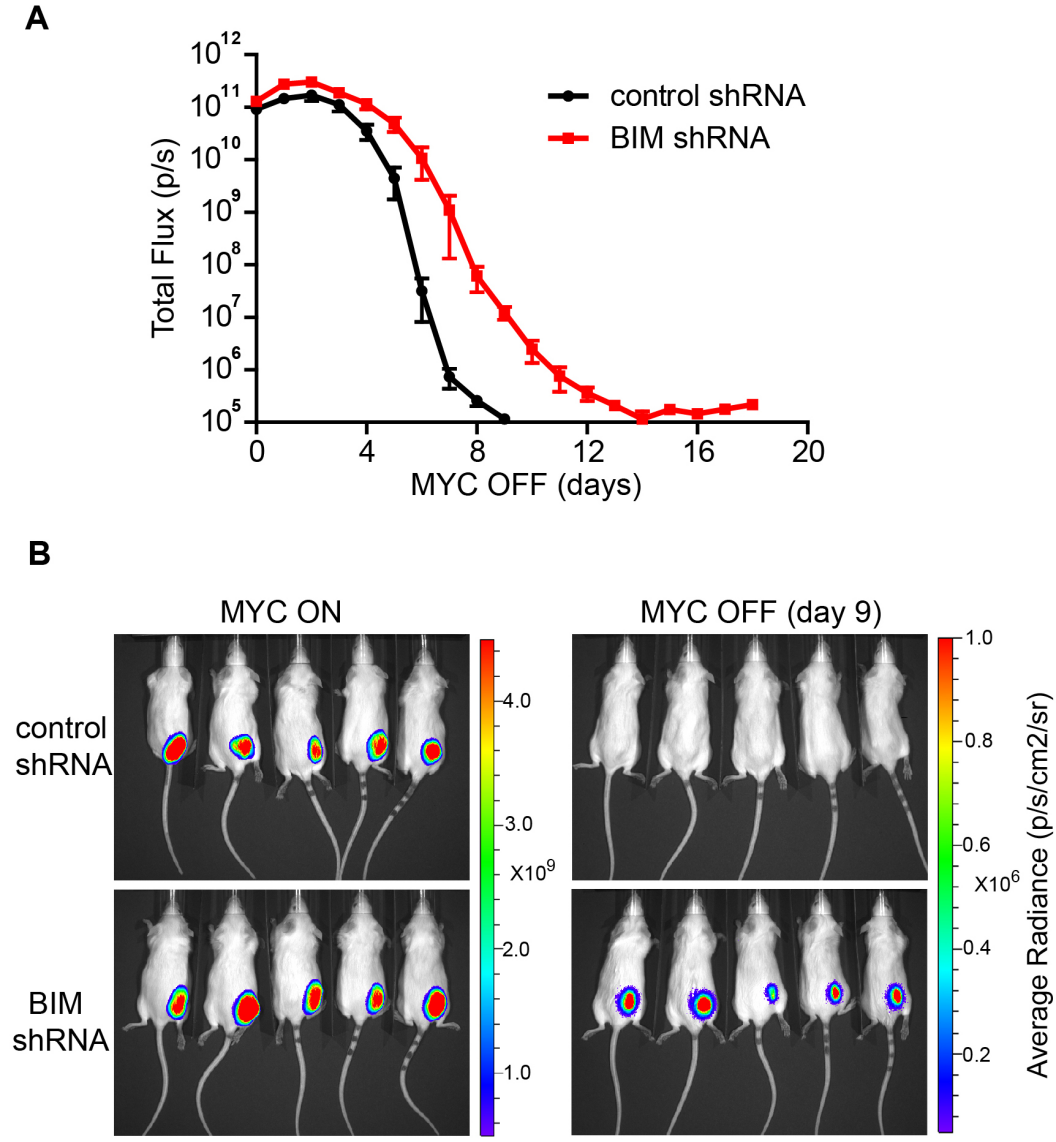
Suppression of BIM expression delays *in vivo* tumor regression upon oncogene inactivation **A.** Kinetics of tumor regression in control *versus* BIM knockdown tumors. The total flux of bioluminescence signal was used to measure the tumor mass. Five mice per group were imaged immediately before doxycycline treatment and then daily after doxycycline treatment for 18 days. The data at each time point were presented and plotted as mean +/− SEM. The kinetics of tumor regression between control and *Bim* shRNA group is significantly different (*p* < 0.05. two-way ANOVA with Bonferroni post-test). **B.** Tumor mass shown by bioluminescence imagining. Both MYC ON tumors and MYC OFF (day 9) tumors are shown. The colored scale bar indicates the range of the average radiance (p/s/cm2/sr) of the tumor area.

We then examined if knockdown of BIM expression influences the kinetics of tumor regression upon oncogene inactivation. The MYC ALL tumor cells were labeled with firefly luciferase and transplanted subcutaneously into syngeneic FVB/N hosts. After tumors were established, oncogene expression was turned off with administration of doxycycline in drinking water. We observed that the kinetics of tumor regression was significantly delayed upon BIM knockdown as shown by bioluminescence imaging (Figure 3A–3B). Thus, our data suggests that BIM is important for robust tumor regression upon oncogene inactivation.

### *miR-17-92* expression blocks the induction of BIM-mediated apoptosis in MYC-, RAS-, but not in BCR-ABL-driven ALLs

**Figure 4:**
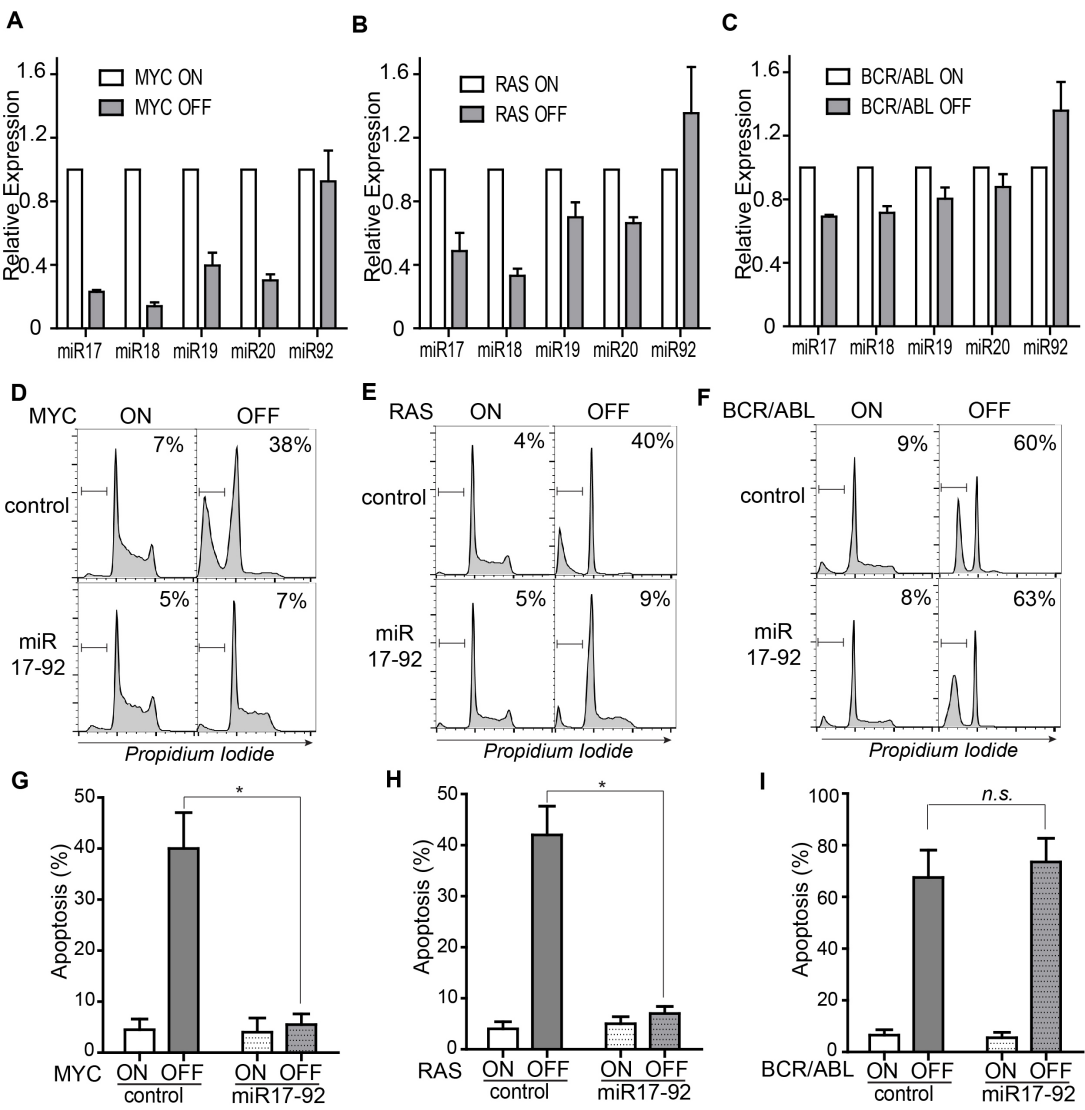
The microRNA cluster, *miR-17-92*, regulates apoptosis in MYC-, RAS- but not BCR-ABL-driven ALL model **A.**-**C.** Expression of individual microRNAs in the *miR-17-92* cluster upon inactivation of the driver oncogenes. The expression levels of each microRNA were quantified using real-time PCR and normalized to expression level of endogenous U6 snRNA. **D.**-**F.** Apoptosis induction and cell cycle distribution in the control and *miR-17-92* expressing tumor cells. Cell cycle distribution was assessed by flow cytometric analysis of propidium iodide-stained cells. Apoptosis was quantified by gating on the sub-G1 populations. Oncogene expression was shut off for three days in the MYC and RAS models and for two days in the BCR-ABL model. The gates for sub-G1 populations are set as indicated. The relative percentage of apoptotic populations is labeled in the top right corner of the plots. For each cell line, only one representative plot is shown. Data for the replicates was further quantified and presented in figure G-I. **G.**-**I.** Quantification of sub-G1 populations in the control and *miR-17-92* expressing tumor cells. Each time point includes at least three replicates. Data are presented as mean +/− SEM. Student's *t* test. *p < 0.001. n.s. stands for Not Statistically Significant.

BIM level is known to be suppressed by *miR-17-92* during normal development of lymphocytes [22, 23]. We speculated that downregulation of *miR-17-92* upon oncogene inactivation may lead to the induction of BIM. We thus examined changes in the expression of individual microRNAs in the *miR-17-92* cluster upon oncogene inactivation in our ALL models driven by MYC, RAS, and BCR-ABL. In both MYC- and RAS-driven ALL models, significant downregulation of *miR-17-92* was observed upon oncogene inactivation (Figure 4A–4B). In contrast, the downregulation of *miR-17-92* in the BCR-ABL-driven leukemia cell lines was modest (Figure 4C).

To delineate whether *miR-17-92* expression could block the induction of apoptosis, we infected the ALL cell lines with Murine Stem Cell Virus containing *miR-17-92* (*MSCV-miR-17-92*) [24]. *miR-17-92* expression blocked the induction of BIM upon MYC inactivation (Supplementary Figure 2). The apoptosis of tumor cells was examined using flow cytometric analysis of cell cycle distribution after propidium iodide staining. *miR-17-92* expression significantly blocked apoptosis as shown by the reduced sub-G1 populations in MYC and RAS but not BCR-ABL-driven tumors (MYC 38% *versus* 5%; RAS 40% *versus* 9%; BCR-ABL 60% *versus* 63% as shown in Figure 4D–4F and further quantified in Figure 4G–4I). Notably, expression of *miR-17-92* also blocked the proliferative arrest induced by inactivation of MYC or RAS (Figure 4D–4E). These findings suggest that *miR-17-92* plays a pivotal role in maintaining the cancer phenotype by sustaining proliferation and survival in MYC and RAS-driven ALLs. In contrast, *miR-17-92* expression does not seem to regulate apoptosis and proliferative arrest in BCR-ABL cells (Figure 4F).

### BIM induction upon BCR-ABL inactivation is correlated with JNK phosphorylation

To explain how BIM is induced in the BCR-ABL model, we tested other potential mechanisms, such as transcriptional stimulation [25, 26] and JNK- or ERK-mediated phosphorylation [27–30]. Transcriptional stimulation was ruled out because BIM mRNA expression was significantly decreased upon BCR-ABL inactivation (Supplementary Figure 3A). The level of phosphorylated ERK remained constant upon BCR-ABL inactivation as revealed by Western blot analysis (Supplementary Figure 3B). However, we did observe increased JNK phosphorylation upon oncogene inactivation in BCR-ABL but not in MYC or RAS-driven ALLs (Supplementary Figure 3B). Thus, we infer that BIM induction in the BCR-ABL model may be regulated post-transcriptionally through JNK phosphorylation.

## DISCUSSION

We used three conditional transgenic mouse models of ALL driven by different oncogenes to demonstrate that BIM activation is the convergent mechanism of apoptosis associated with oncogene addiction. Interestingly, the mechanism of BIM activation was different depending on the specific driver oncogene. The microRNA cluster, *miR-17-92,* regulates BIM induction and apoptosis in MYC- and RAS- but not in BCR-ABL-driven ALLs. The regulation of BIM-mediated apoptosis in BCR-ABL-driven ALL appears to be post-transcriptional and related to JNK phosphorylation.

Multiple prior studies have correlated BIM activation with clinical response to targeted therapies. Lung adenocarcinoma cells treated with gefitinib, breast cancer cells treated with lapatinib, and melanoma cells treated with B-RAF inhibitors all exhibit BIM activation. In these solid tumors, BIM is often activated *via* inhibition of the MEK/ERK pathway and/or the PI3K/mTOR pathway [10, 11, 15, 31–33]. Our research highlights the critical role of microRNA *miR-17-92* and/or JNK phosphorylation in regulating BIM-mediated apoptosis upon oncogene inactivation particularly in the leukemia contexts. Thus, our work provides novel insight into the mechanism of apoptosis associated with oncogene addiction.

The activity of BIM seems to be particularly critical in regulating the balance between pro-apoptotic and pro-survival signals. Even modest changes in BIM activity can significantly influence tumorigenesis and therapeutic responses of cancer. For examples, the loss of one allele of BIM is sufficient to substantially accelerate lymphomagenesis [34, 35]. Epigenetic silencing of BIM by promoter hypermethylation mediates tumor chemoresistance in Burkitt's lymphoma [36]. An intronic deletion polymorphism that produces a less active form of BIM in chronic myeloid leukemia and lung adenocarcinoma patients leads to therapeutic resistance to tyrosine kinase inhibitors [14, 18]. Thus, BIM is a central mediator of apoptosis associated with therapeutic responses of cancer. Our results support the notion that induction of BIM-mediated apoptosis may be an effective therapeutic option. In particular, activation of BIM-mediated apoptosis pathway using BH3 mimetics may synergize with targeted therapeutics to induce sustained tumor regression [17, 37–41].

## MATERIALS AND METHODS

### Cell culture

The murine ALL leukemia cell lines derived from *Eμ-tTA/TRE-MYC*, *Eμ-tTA/TRE-KRAS*, and *Eμ-tTA/TRE-BCR/ABL* mice were cultured in RPMI-1640 media (Gibco) supplemented with 10% fetal bovine serum under standard culture condition. Inactivation of the Tet-regulated oncogenes was achieved with doxycycline treatment (20ng/ml) in the culture media.

### Western blot and antibodies

Western blots were performed as described before [24]. The following antibodies were used for Western blot: MYC (9E10, EMD Biosciences), Tubulin (Sigma), BIM, phospho-JNK, phospho-ERK, cleaved-caspase 3, and other BCL-2 family proteins (Cell Signaling). For optimizing Western blot images for presentation, only brightness or contrast was adjusted uniformly over the entire image and images represent the original data.

### BIM shRNA knockdown and retroviral expression of *miR-17-92*

The knockdown of BIM expression was accomplished using LMP *miR-30*-based shRNAs with puromycin selection marker (V2LMM_220682 from Open Biosystems). Retrovirus was prepared using the phoenix retrovirus packaging system. The leukemia cells were spin infected and selected with puromycin (2μg/ml).

### Bioluminescence imagining of *in vivo* tumor regression

The tumors cells were labeled with MSCV-luciferase and transplanted subcutaneously into FVB/N mice. When tumors reach approximately 1.0cm in diameter, mice were imaged and then treated with doxycycline (20μg/ml). For imaging procedure, the mice were injected intraperitoneally with 100μl of d-luciferin (33mg/ml) about ten minutes before detection using the IVIS 200 cooled CCD camera system (Xenogen). Mice were imaged daily for 18 days following doxycycline treatment. Quantification of bioluminescence signals was done using Living Imaging software 4.3.1 (Perkin Elmer). All animal experiments were approved by Stanford's Administrative Panel on Laboratory Animal Care (APLAC) and were performed in accordance with institutional and national guidelines.

### Quantification of Bim mRNA and *miR-17-92* expression

*Bim* mRNA expression was quantified in triplicates using SYBR based quantitative PCR and normalized to Ubc mRNA level. Primers used are: *Bim*-F 5′-CACCTGCTGTGTGCTTCCTA-3′ and *Bim*-R 5′-TTCAGTGAGCCATCTTGACG-3; *Ubc*-F 5′-AGCCCAGTGTTACCACCAAG-3′ and *Ubc*-R 5′-ACCCAAGAACAAGCACAAGG-3′. Total microRNA was extracted with miRNeasy microRNA extraction kit (Qiagen). The microRNAs were first reverse-transcribed and then quantified with TaqMan microRNA assay kits (Applied Biosystems) following manufacturer's protocols. Each sample was run in triplicate and normalized to U6 snRNA.

### Flow cytometric analysis of cell death

Propidium iodide staining was used for the study of cell cycle distribution and apoptosis. Briefly, leukemia cells were resuspended in PBS and fixed with ice-cold ethanol. Cells were treated with RNase and propidium iodide and analyzed on a FACScan flow cytometer (Becton Dickinson). For live/dead staining, cells were stained with 7-AAD. FACS data was analyzed with FlowJo software (Tree Star).

## SUPPLEMENTARY MATERIAL FIGURES


