# Supplementary material for: BIM mediates oncogene inactivation-induced apoptosis in multiple transgenic mouse models of acute lymphoblastic leukemia
Source: Oncotarget. 2016 Apr 14;7(19):26926–34. doi: 10.18632/oncotarget.8731 (PMC5053622; doi:10.18632/oncotarget.8731)
Supplement: Supplementary file 1 [file oncotarget-07-26926-s001.pdf]

BIM mediates oncogene inactivation-induced apoptosis in multiple transgenic mouse models of acute lymphoblastic leukemia

Supplementary Material

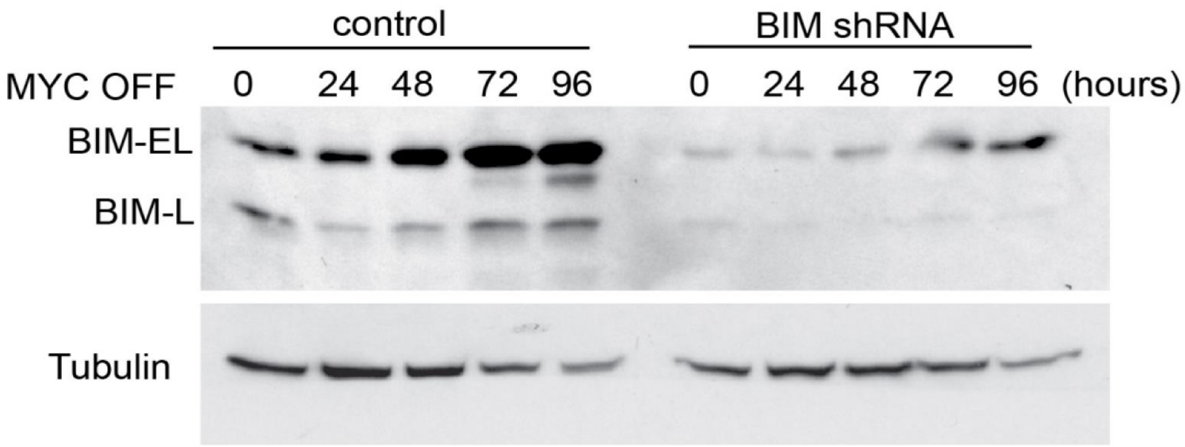

Supplementary figure 1. Efficiency of *Bim* shRNA knockdown shown by Western blot analysis. MYC expression was shut off in the MYC-driven ALL cells for four days. Samples were taken daily and submitted to Western blot analysis.

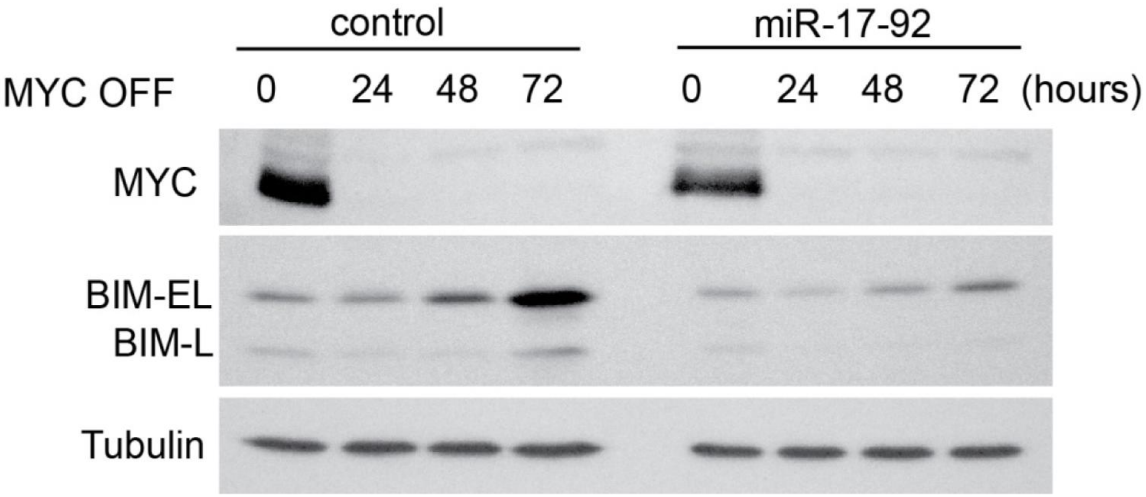

Supplementary figure 2. Downregulation of BIM expression by *miR-17-92* in MYC-driven ALL tumor cells. MYC expression was shut off for three days. Protein samples were taken daily and submitted to Western blot analysis.

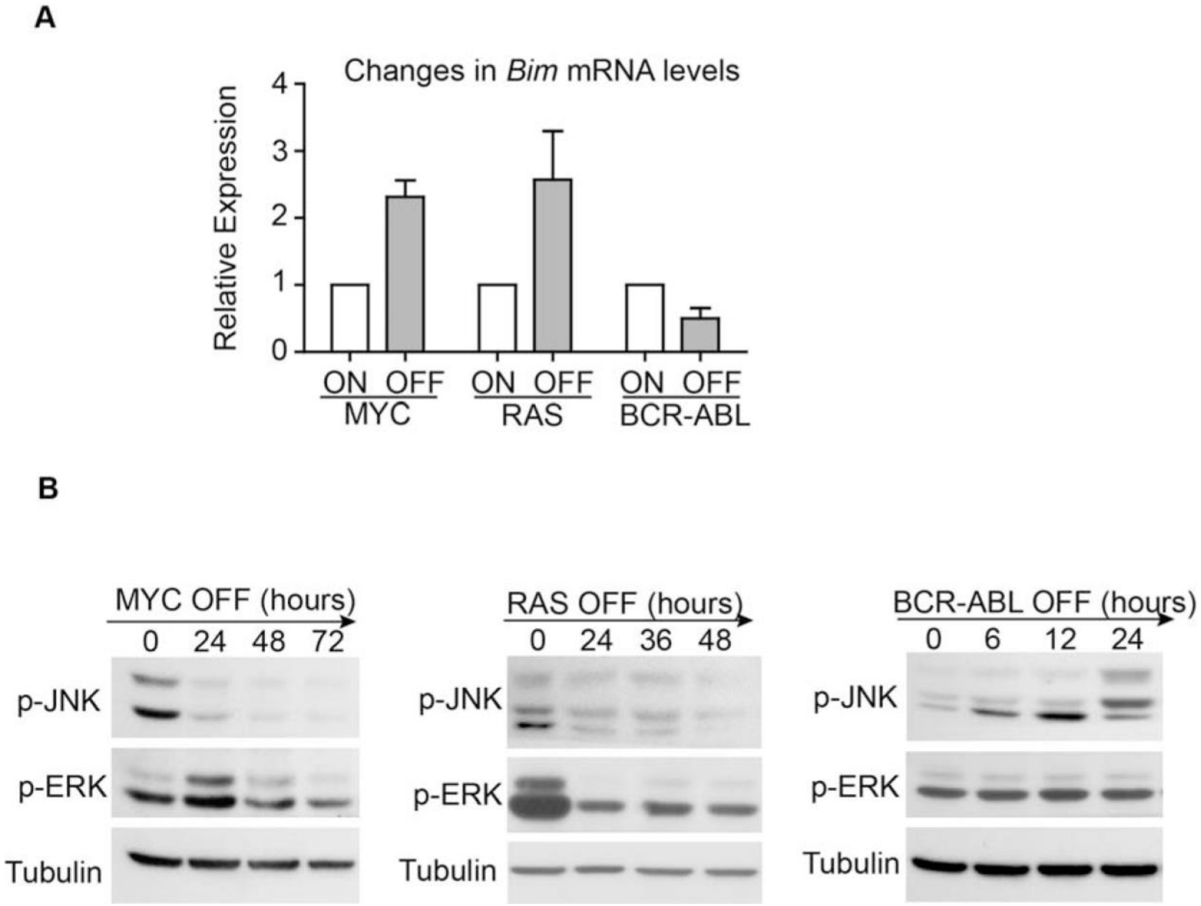

Supplementary figure 3. BIM activation via posttranscriptional regulation upon BCR-ABL inactivation. A.) Change in *Bim* mRNA level upon oncogene inactivation in three ALL models. Expression of the oncogene is shut off for three days in the MYC and RAS models and for two days in the BCR-ABL model. **B.**) Analysis of JNK and ERK phosphorylation by Western blot upon inactivation of the respective driver oncogene. Tubulin serves as the loading control.
